# The search for assisted reproduction: profile of patients seen in the fertility outpatient clinic of a public hospital

**DOI:** 10.5935/1518-0557.20200008

**Published:** 2020

**Authors:** Fernanda Polisseni, Miralva Aurora Galvão Carvalho, Gabriel Duque Pannain, Leda Caldeira de Souza, Valeska Alice Teixeira de Oliveira

**Affiliations:** 1 Surgery Department, Medical School - Federal University of Juiz de Fora, Juiz de Fora, MG, Brazil; 2 Federal University of Juiz de Fora, Juiz de Fora, MG, Brazil

**Keywords:** Infertility, reproductive health, causes of infertility

## Abstract

**Objective:**

To analyze the epidemiological profile of patients treated at the Fertility Outpatient Clinic of a tertiary public hospital in Juiz de Fora.

**Methods:**

This cross-sectional study analyzed the medical records of 448 patients who sought fertility treatment at a tertiary public hospital. The data collected from the medical records were used to assess the main causes of infertility, find the most frequently performed procedures, and the cases eligible to therapeutic or prophylactic intervention.

**Results:**

Of the 448 patients included in the study, 385 (86%) sought fertility consultation, 49 (10%) came in for repeated miscarriages, and 14 (3%) for other reasons. Of the 438 infertile patients, 280 (63.9%) had primary and 158 (36.1%) had secondary infertility. The top-three conditions of the 295 patients with established diagnoses were chronic anovulation (n=98; 33%); tubal factor infertility (n=86; 29%); and male factor infertility (n=59; 20%).

**Conclusions:**

Improving care in reproductive health requires a more profound comprehension of the epidemiological profile of patients seeking treatment. There are alternative cost-effective means to contain the development of infertility. Additional expenditure in public healthcare is needed to accommodate the growing number of individuals seeking fertility treatment in Brazil.

## INTRODUCTION

Infertility is characterized by failure to achieve clinical pregnancy after one year or more of regular unprotected sexual intercourse (Saraçol *et al*., 2017). Infertility is categorized as primary when the existence or prior pregnancies cannot be proven or as secondary when there is reliable record of at least one past pregnancy (Olooto *et al*., 2012). Recent studies estimated the prevalence of infertility in the population to range between 9% and 18%. In infertile couples, 35% of the cases are caused by female factors, 35% by male factors, 20% by combined factors, and 10% by unknown reasons (Aghajanova *et al*., 2017; Graner & Barros, 2009; Díaz Bernal & García Jordá, 2010; Faria *et al*., 2012).

Investigation is indicated for couples unable to achieve pregnancy after one year of regular unprotected sexual intercourse. Women aged 35 years or older and individuals with known comorbidities associated with infertility unable to achieve pregnancy after six months of regular unprotected sexual intercourse are also good candidates for additional investigation. Infertility investigation must include the members of the couple and comprise detailed interviews, physical examination, and specific tests. Psychosocial factors must also be considered (Carvalho *et al*., 2016). Other essential items in infertility investigation are ovarian function and structure, patency of the female genital tract, uterine condition assessment, and semen analysis.

A significant proportion of cases of female infertility have been related to advanced age (35+ years). Tubal and peritoneal factors are quite frequent in Brazil, mainly due to sexually transmitted diseases (STD). Infertility risk factors also include endometriosis, uterine myomatosis, chronic diseases, and use of medication (Dias *et al*., 2014).

In Brazil, family planning is enforced via Law 9263 enacted on January 12, 1996. Actions in this area include fertility regulation and protection of the right to constitute a family and control the number of children in a family - not quite as in birth control, in which the State intervenes solely to decrease birth rates (Silva *et al*., 2009). Although parents have the right to have as many children as they like, health policies related to family planning revolve around contraception, while services that have policies related to infertility are rare (Dias *et al*., 2014).

An estimated 3.6 million women rely solely on the Brazilian public healthcare system (SUS, Brazilian acronym) for fertility treatment. However, the public healthcare system is currently unable to cope with the needs of these patients (Souza, 2014). This situation demonstrates the magnitude of this public healthcare issue and underlines the importance of improving access to services that offer diagnostic support and fertility treatment, including the high complexity procedures of assisted reproduction, which are even rarer within the scope of the SUS. Most of the just over 100 reproductive health centers in Brazil are private clinics. In vitro fertilization is costly, which makes treatment inaccessible to a great portion of the interested population (Corrêa & Loyola, 2015). Approximately 5% of the individuals in need of fertility treatment are seen at public clinics, with 300 to 1,500 patients in their waiting lists and waiting times that may range from six months to four years (Corrêa & Loyola, 2015; Souza, 2014).

Since the first baby born from in vitro fertilization in 1978, more than 8 million children have been brought into the world via assisted reproduction techniques. This data demonstrates the importance and growth of the number of women undergoing ART procedures. Knowing the profile of patients that require these services is paramount, as it allows the structuring and development of effective public initiatives to improve reproductive healthcare.

## MATERIAL AND METHODS

This cross-sectional qualitative descriptive observational retrospective study included the medical records of 448 patients treated at the Fertility Clinic of a tertiary public hospital in the Brazilian southeastern city of Juiz de Fora between January 1, 2015 and December 31, 2018. Patients were interviewed in the first visit. We analyzed the notes in the medical records referring to every patient’s first and last consultation at the service.

Patient medical charts were analyzed for epidemiological data including age; occupation; smoking; drinking and lifestyle (sedentary *vs.* active). Patient partners were analyzed for age; occupation; comorbidities; number of children; smoking and drinking. Patients were also analyzed for general gynecological data, including items such as age of menarche; menstrual cycle characteristics; presence and intensity of dysmenorrhea; age of coitarche; number of partners; presence of dyspareunia; bleeding during intercourse; frequency of sexual intercourse; perception of ovulation signs; number of pregnancies, births and previous abortions.

The patients in the group that consulted with a physician for infertility was analyzed for time of unprotected intercourse; primary or secondary infertility; prior treatment; cause of infertility and treatment instituted. The study was submitted to the Local Ethics and Research Committee and approved under opinion report number 3.233.999 and Certificate of Presentation for Ethical Presentation (CAAE) 97931118.1.0000.5133.

Data was organized into *Excel for Windows* spreadsheets and analyzed by descriptive statistics with the aid of the *Statistical Package for the Social Sciences* - SPSS, version 18.0.

## RESULTS

The analysis of patient medical records fleshed out the main causes of infertility in the area served by the hospital, the most commonly performed procedures, and potential targets for specific therapeutic and prophylactic interventions.

The medical records of 448 patients seen in a fertility outpatient clinic were distributed as follows: 385 (86%) patients sought infertility consultation; 49 (10%) came in for repeated miscarriages; and 14 (3%) for other reasons, including endocrine disorders and referral to another service.

Of the infertile 438 patients, 280 (63.9%) had primary and 158 (36.1%) had secondary infertility. In addition, 114 (26.5%) of the 385 patients who ranked inability to achieve pregnancy as their main concern had sought care or undergone treatment prior to the dates covered in the study. Patients attempted to get pregnant for 4.69 years (4.31-5.06; 95% confidence interval) on average before seeking fertility care, with the shortest reported time being one year and the longest 23 years.

The mean age of the patients seeking consultation was 32.68 years (31.98-33.39; 95% confidence interval), with the lowest age being 18 and the highest 51 years. The mean age of their partners was 33.74 (32.66-34.82; 95% confidence interval), with the youngest being 20 and the oldest 61 years old. When assessed for occupations with increased risk of infertility, only three (1.3%) patients and 60 (18.2%) partners had jobs in which they were exposed to risk of infertility. In addition, 110 partners (30.8%) had comorbidities potentially related to changes in sperm production such as varicocele, history of testicular torsion, and orchitis. Lifestyle analysis revealed that 46 (12%) were smokers and 86 (22%) drank alcohol. Physical activity was a habit for 80 (21%) patients, with a minimum of three sessions of exercises a week.

The following variables were analyzed from the gynecological and obstetric history of the patients: 204 (45.5%) patients had regular menstrual cycles; 148 (33.0%) reported irregular menstrual cycles; and 96 (21.4%) were unable to answer this question. Dysmenorrhea, which was reported by 204 (45.5%) women, was described in 65 (27.7%) cases as being intense or disabling, while 86 (38.6%) had to take symptom-relief medication. Thirty patients (9.1%) had frequent post-intercourse bleeding, while eight (2.4%) had sporadic events. In addition, 107 (30.3%) women reported dyspareunia. Fifteen patients (3.4%) had undergone tubal ligation and had a desire to become pregnant.

Diagnostic information was cited in 295 of the 448 patient charts analyzed. Ninety-eight patients (33%) were diagnosed with chronic anovulation; 86 (29%) with tubal factor; 59 (20%) with male factor; 63 (21%) with uterine factor; 13 (4%) with infertility without apparent cause; 28 (9%) with repeated miscarriages; and eight (3%) with infertility for other causes, including four with gonadal dysgenesis, three homosexuals; and one with extra-vaginal ejaculation.

The patients in the group with chronic anovulation had the condition due to polycystic ovary syndrome (n=20; 20%); premature ovarian failure (n=8; 8%); hypothyroidism (n=7; 7%); advanced age (n=6; 6%); hyperprolactinemia (n=5; 5%); or hypothalamic amenorrhea (n=1; 1%). The cause of anovulation was undefined in 51 individuals (51%). Forty-seven individuals (55%) in the group of patients with tubal factor infertility had endometriosis.

Information on patient management was recorded in 256 medical records, since additional care measures were defined only after the patients came back to the service with the results from the ordered tests. Eighty-four patients (33%) underwent ovulation induction and timed intercourse (treatment offered at the university hospital in question). Nine (11%) became pregnant by the end of this study. Twelve patients (5%) were referred to the endocrinology department for endocrine disorders; 44 couples (17%) were referred to specific urological evaluation; 73 patients (29%) underwent videolaparoscopy to treat tube-peritoneal factor infertility (treatment offered at the university hospital in question); 90 (35%) were referred to IVF or IUI (at another service); and ten (4%) were referred to the hematology service.

## DISCUSSION

Infertility is a complex condition with significant medical, psychosocial, and economic repercussions (Kuohung & Hornstein, 2013). Effective public healthcare strategies focused on primary prevention might decrease the prevalence of infertility, improve reproductive health and quality of life, and reduce treatment costs. Primary prevention - including screening for and treating females with Chlamydia infection along with patient education on sexually transmitted diseases - might be an option for some causes of infertility. Other cost-effective measures with proven efficacy at maintaining good reproductive health include anti-smoking campaigns directed to adolescents and young adults, physical activity, and healthy diets (Macaluso *et al*., 2010; Coffield *et al*., 2001).

Our study found a higher proportion of individuals with primary infertility, as described in the literature (Masoumi *et al*., 2015; Karimpour Malekshah *et al*., 2011). Patients attempted to become pregnant for 4.69 years on average before seeking medical help, a longer period of time than the average reported in studies carried out in other developing countries (Masoumi *et al*., 2015; Nwajiaku *et al*., 2012). A 2010 study enrolling women from France and England found that female reproductive history was a strong factor in determining the time for which women would wait before seeking medical help. Nulliparous women were twice as likely to seek help compared to women who already had children, suggesting that these couples were less concerned with their future fertility. Patients with a history of gynecological disorders also sought specialized care earlier. Our study found that schooling was a factor independent from gynecological history, with women of higher schooling seeking fertility care earlier (Moreau *et al*., 2010).

A factor of considerable importance is the long time it takes for patients to schedule medical appointments with physicians from specific medical specialties on account of the limited number of specialist physicians in public health services (Makuch *et al*., 2010). Studies showed that patients wait for six months on average for consultations with specialized physicians in the Brazilian public health system (Makuch *et al*., 2011). And when they finally see a doctor, most of the patients are unable to bear the costs of treatment, medicines, supplies, and operating expenses (Makuch *et al*., 2010). Only 108 (28%) of the 385 patients seeking consultation for infertility had sought medical care or undergone treatment, most of which at private clinics, a number considerably lower than the ones reported in the literature (Gradvohl *et al*., 2013).

Age is a key factor in attaining spontaneous conception and conception via treatment, with gradual declines in fertility occurring mainly after 35 years of age (Saraçol *et al*., 2017). The patients seen in our fertility outpatient clinic were aged 32.68 years on average, which places them within the ideal age range to conceive. However, the reality our patients live in dictates that they often take longer to seek and access care; and care outcomes may be negatively affected by delays in performing tests and starting treatment.

Women suffer from reproductive capacity decline at an earlier age. Men, by their turn, maintain reproductive capacity for longer, with progressive declines starting after the age of 40 years and gaining momentum after 50 years of age. Comorbidities such as varicocele, cryptorchidism, primary ciliary dyskinesia, and sexually transmitted diseases may also interfere with reproductive sexual health (Hamada *et al*., 2016; Carrell *et al*., 2016; Fode *et al*., 2016).

Smoking is a known risk factor to reproductive health on account of its deleterious effects on conception, ovarian follicular dynamics, sperm parameters, and mutations induced in gametes, in addition to impacts on outcomes after reproductive medicine therapy. The magnitude of the impact of smoking on infertility has been associated with time of smoking, number of cigarettes smoked a day, and total consumption of cigarettes (Yang *et al*., 2017). Although cigarette smoking is a known risk factor, 46 patients (12%) were smokers. This finding reinforces the need to strengthen patient advice and introduce preventive measures to individuals of younger ages. Fortunately, studies indicated that former smokers have similar fertility rates compared with individuals who never smoked, suggesting that the effects of smoking can be reversed (Rossi *et al*., 2016).

Unlike smoking, the correlation between drinking and infertility is controversial in the literature. Alcoholism has been strictly described as a proven cause of sexual impotence and teratogenicity (Schaeffer & Hotaling, 2016). Regular physical activity is a protective factor for infertility (Rossi *et al*., 2016). However, only 80 patients (21%) performed exercises regularly, which reaffirms the need to strengthen the preventive advice given not only to patients seeking care for infertility, but to individuals seen in all areas of medicine.

In terms of reported complains, 204 patients (45.5%) had dysmenorrhea; 65 of them (27.7%) had severe dysmenorrhea. The relative risk of endometriosis occurring with dysmenorrhea was 1.39 (95% confidence interval; 1.16-1.68), thus supporting the association previously described in the literature.

Sterilization was once the most widely used contraceptive method in Brazil. However, two of the most recent national demographic and health surveys conducted in the country in 1996 and 2006 showed that the prevalence of tubal ligation decreased from 40% to 29% (Ministério da Saúde, 2008; BEMFAM, 1997). National demographic data undergoing the procedure (Nicolau *et al*., 2011). A 2017 study showed that 12-15% of the patients submitted to tubal ligation regretted or were unhappy with the procedure (Eskicioglu *et al*., 2017). Although the rates in the literature are considerably higher than the rate found in our study (3.4%), it should be mentioned that the aforementioned procedure is only performed in our center after the patients have been extensively informed of its potential consequences, which mitigates the chances of dissatisfaction.

Similarly to the literature, anovulatory disorders ranked atop the list of causes of infertility in our study (Kazemijaliseh *et al*., 2015). Ovarian conditions were the main cause of anovulatory disorders in the literature and in our study, mostly polycystic ovary syndrome and premature ovarian failure, diseases with a significant genetic influence (Anwar & Anwar, 2016). Tubal and uterine factor infertility stood out from other conditions in our analysis, as also observed by other authors (Briceag *et al*., 2015). As reported in the literature, the most prevalent etiology of tubal factor infertility in our study was endometriosis. Other authors reported lower quality oocytes and embryos in patients with endometriosis and an association with increased difficulty to perform implantation and achieve fertilization (Navarro *et al*., 2003).

More than 60% of the patients included in our study required high complexity procedures - videolaparoscopy and in vitro fertilization - often unavailable in public health centers. There are only 14 hospitals in the Brazilian public health system with sufficient resources to perform *in vitro* fertilization. In these centers, patients wait for six months to four years for a procedure (Souza, 2014). Although 35% of the patients have been referred to IVF or IUI, the chances of having the procedure done in a public hospital are minimal, with long waiting lines and, consequently, significant psychological, familial, and social distress. Despite legislation granting universal access to these medical services in the public health system, assisted reproductive technologies are mostly confined to private centers for economic reasons (Corrêa & Loyola, 2015).

Fertility treatment is costly in terms of money, time, and emotional expenditure (Rossi *et al*., 2016). Treatment success has been tied to myriad factors. Therefore, we should strive to address the factors under the control of our patients, including measures such as encouraging physical activity and smoking cessation for their relative simplicity and impact on reproductive health (Rossi *et al*., 2016).

## CONCLUSION

Improving care in reproductive health requires a more profound comprehension of the epidemiological profile of patients seeking treatment. Other cost-effective measures with proven efficacy at maintaining good reproductive health include anti-smoking campaigns directed to adolescents and young adults, physical activity, and healthy diets.

Infertility has far-reaching effects in the lives of affected individuals, and a large part of the Brazilian population cannot afford fertility treatment. Additional expenditure in public healthcare is needed to accommodate the growing number of individuals seeking fertility treatment in Brazil.
